# Southampton mealtime assistance study: design and methods

**DOI:** 10.1186/1471-2318-13-5

**Published:** 2013-01-07

**Authors:** Helen C Roberts, Anna L Pilgrim, Marinos Elia, Alan A Jackson, Cyrus Cooper, Avan Aihie Sayer, Sian M Robinson

**Affiliations:** 1 Academic Geriatric Medicine, University of Southampton, Southampton General Hospital, Tremona Road, Southampton Hampshire, SO16 6YD, UK; 2 MRC Lifecourse Epidemiology Unit, University of Southampton, Southampton General Hospital, Tremona Road, Southampton Hampshire, SO16 6YD, UK; 3 Southampton NIHR Biomedical Research Centre in Nutrition, Diet and Lifestyle, University Hospital Southampton NHS Foundation Trust, Southampton, SO16 6YD, UK

**Keywords:** Nutrition, Older, Volunteer, Mealtime assistance, Dietary intake, Hospital

## Abstract

**Background:**

Malnutrition is common in older people in hospital and is associated with adverse clinical outcomes including increased mortality, morbidity and length of stay. This has raised concerns about the nutrition and diet of hospital in-patients. A number of factors may contribute to low dietary intakes in hospital, including acute illness and cognitive impairment among in-patients. The extent to which other factors influence intake such as a lack of help at mealtimes, for patients who require assistance with eating, is uncertain. This study aims to evaluate the effectiveness of using trained volunteer mealtime assistants to help patients on an acute medical ward for older people at mealtimes.

**Methods/design:**

The study design is quasi-experimental with a before (year one) and after (year two) comparison of patients on the intervention ward and parallel comparison with patients on a control ward in the same department. The intervention in the second year was the provision of trained volunteer mealtime assistance to patients in the intervention ward. There were three components of data collection that were repeated in both years on both wards. The first (primary) outcome was patients’ dietary intake, collected as individual patient records and as ward-level balance data over 24 hour periods. The second was clinical outcome data assessed on admission and discharge from both wards, and 6 and 12 months after discharge. Finally qualitative data on the views and experience of patients, carers, staff and volunteers was collected through interviews and focus groups in both years to allow a mixed-method evaluation of the intervention.

**Discussion:**

The study will describe the effect of provision of trained volunteer mealtime assistants on the dietary intake of older medical in-patients. The association between dietary intake and clinical outcomes including malnutrition risk, body composition, grip strength, length of hospital stay and mortality will also be determined. An important component of the study is the use of qualitative approaches to determine the views of patients, relatives, staff and volunteers on nutrition in hospital and the impact of mealtime assistance.

**Trial registration:**

Trial registered with ClinicalTrials.gov NCTO1647204

## Background

Malnutrition is common in older people [1]. For example, in a recent pooled analysis of data from 4,500 older individuals across 12 developed countries (mean age 82 years), 23% were identified as malnourished using the Mini Nutritional Assessment, with a further 46% at risk of poor nutrition [2]. However, there were marked differences in prevalence of malnutrition according to setting, reaching 39% in hospitalised older patients. Although malnutrition is known to be associated with major adverse clinical outcomes such as increased mortality [3], morbidity [1] and length of stay [4] at enormous cost to individuals and health services, and despite current prevalence estimates, it still remains an undertreated problem [5].

One consequence of the estimated rates of malnutrition among older hospitalised patients is that concerns have been raised about hospital food, both in terms of the quality of food provided as well as adequacy of provision of assistance with eating at mealtimes [6,7]. But it is important to recognise that there are a number of other contributors to low dietary intake among older patients, which include the effects of acute illness on appetite, cognitive impairment, low mood, co-morbidities and altered taste sensation due to illness and medications, each of which may predate admission to hospital [8,9]. Thus while malnourished older adults may be disproportionately represented in hospital and much of the evidence has come from hospital settings [3], malnutrition is largely found and originates in the community [5,10].

However, it remains a concern that low energy and nutrient intakes and poor nutrient status are commonly described in older hospitalised patients [9,11,12]. Whether provision of more effective support at mealtimes, including assistance with eating, could improve intake remains uncertain. In the UK, the standard of mealtime care in hospitals has been an issue of concern for a number of years. A report in 2008 from the Healthcare Commission found that one in five patients who wanted help eating did not get it [13]. More recently, the Care Quality Commission reported that 15% of 100 hospitals surveyed in 2011 were not meeting the minimum legal standards for mealtime care in the elderly, and another 32% needed to improve [14]. This problem is not unique to the UK and has been reported in other countries such as Australia and the USA [15,16].

Mealtime assistance has been defined as the process of enabling a person to complete the eating process when a meal or snack is served in a care setting [17]. This assistance can range from presentation of food in an available form and providing verbal encouragement, to cutting food into smaller pieces and transferring food from the plate to the person’s mouth. Mealtime assistance has been identified as a possible means of improving dietary intake as 70-80% of older in-patients are estimated to require help [18]. However the use of volunteer mealtime assistants has been little researched and their effectiveness is unknown [19].

Two UK research studies of the use of volunteers to help with mealtimes have found conflicting results. A study of 318 older women aged 65 years and over with hip fracture found that additional help from one health care assistant led to increased energy and nutrient intakes and improved clinical outcomes [20]. However a similar study on acute care of the elderly wards, which involved a more heterogeneous group of 592 men and women aged 65 years and over with a range of medical conditions in a randomised controlled trial, found no increase in dietary intake when one healthcare assistant per ward was available to help with eating at two meals on five days per week [21].

The Southampton Mealtime Assistance Study was established in 2010, with the aim of investigating whether the use of trained volunteers employed specifically to focus on mealtime assistance can increase the dietary intake of older acute medical in-patients.

## Methods/design

### Study design

The study design is quasi-experimental [22,23] with a before (year one) and after (year two) comparison of patients on an intervention ward and parallel comparison with patients on a control ward in the same department. The intervention in the second year was the provision of trained volunteer mealtime assistance to patients in the intervention ward.

**Figure 1:**
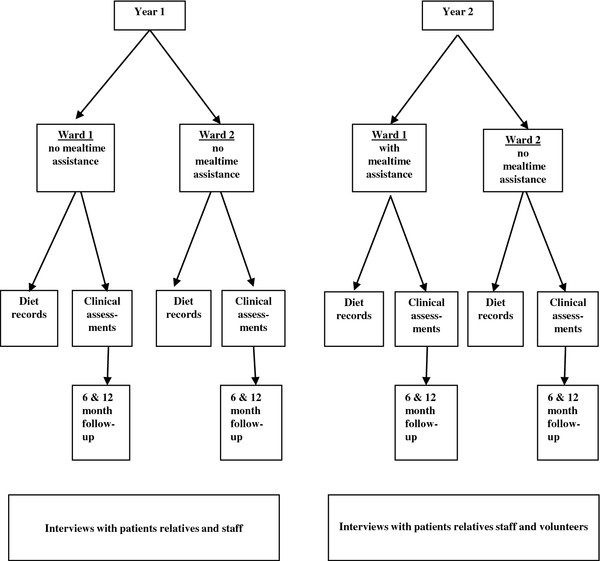
Study outline scheme.

There were three components to the data collection, which were repeated in year one and year two (Figure 1). The primary outcome was dietary intake of patients, collected as individual patient records and ward-level balance data for both wards over 24 hour periods in both years. The second component was clinical outcome data assessed on admission and discharge from both wards and at follow-up 6 and 12 months later. Finally, qualitative data on the views and experience of patients, carers, staff and volunteers was collected through interviews and focus groups in both years to allow a mixed-method evaluation of the intervention [24]. The study received full approval by the local research ethics committee.

### Setting

The study was conducted on two acute wards in the Medicine for Older People Department at a university hospital in England. Medical and nursing care on both wards were very similar, and they had a similar case mix since both admitted acutely unwell female patients aged 70 years and over direct from the Acute Medical Unit and Accident and Emergency Department to the next available bed.

### Participants

All patients admitted to these wards as an emergency between February 1^st^ 2010 and January 31^st^ 2012 were eligible for the study. Exclusion criteria included patients who were nil by mouth, receiving tube feeds, under 70 years old, male, on the Liverpool Care Pathway for the dying patient or being nursed in side rooms.

### Intervention

The intervention was the provision of several trained volunteers to assist at mealtimes. Assistance was given by volunteers who had completed a half day hospital training programme run by speech and language therapy, dietetics and voluntary services. The training included aspects of nutrition in older in-patients, the role of the volunteer, special diets, safe feeding strategies and completion of food and fluid charts. Volunteers were available to assist at weekday lunchtimes. Patients needing help were identified by a senior nurse and assistance included encouragement, opening packages and cutting up food, physical help with getting spoons ready and help guiding the spoon to the mouth as required. Volunteers did not assist patients with known swallowing problems or who were drowsy or unable to sit up.

### Data collection

#### 1. Dietary data collection

The dietary intake of all eligible patients on the wards was studied for 24 hour periods on 14 weekdays between April and December 2010 in year one, and on 12 weekdays between April and November 2011 in year two. The spread of study days captured seasonal variation in menus and illness patterns. The intake of individual patients was recorded at three mealtimes and seven drinks rounds, and for sip feeds, extra food and drinks (such as those brought in by relatives) and any additional food or drinks taken during the night. Research nurses recorded all food items delivered to the patients and collected the trays and cups at the end of each mealtime and drinks round. All food and drinks that were not consumed were weighed and recorded. Sip feeds were collected and weighed at intervals during each study day and research nurses recorded in detail all additional food and drink consumed by individual patients.

##### Calculation of nutrient intake

The nutrient intake of individual patients was calculated from the weight of food consumed and its nutrient content. For main meals the weight of food delivered to each patient was obtained from the hospital caterers, whose portion control measures ensured that each meal component was within 10% of the stated weight. For other foods, standard portion sizes [25] and manufacturers’ information was used. Weights of servings of porridge, soup and custard were determined from the average weights of test portions served in the cups and bowls used on the wards. Standard protocols were devised for converting leftover drinks into the constituent components according to the size of cup and type of milk used, and for the separation of uneaten mixed milk and breakfast cereal. Where other uneaten foods were mixed together, e.g. potato and gravy, and could not be weighed separately, the combined weight of the mixture was recorded and the amount consumed was calculated according to the proportion of each component served in the original meal. Sip feed containers were weighed and the amount consumed calculated. The weighing scales used were calibrated three monthly and accurate to ±0.1 g.

All data were computerised and the computer records of food type and weight were checked in detail for each patient by a single researcher (SR), who was blinded to the status of the wards. Nutrient intake for each patient was calculated from the weight of each food multiplied by its nutrient composition, and summed. Food composition data was obtained from the hospital caterer and the UK food composition database.

##### Additional data collection on dietary study days

Each patient’s height, weight, ‘Malnutrition Universal Screening Tool’ (‘MUST’) score [26] and Body Mass Index (BMI) was abstracted from their clinical records. Height was derived from information given by the patient or calculated using ulnar length [27]. Weight was measured using the ward chair scales. On the study days the date of birth and current primary diagnosis were abstracted from patients’ clinical records. The level of mealtime assistance received at lunchtime by each patient was observed and recorded as follows: 0 = none, 1 = cutting and preparation, 2 = encouragement, 3 = feeding, 4 = refused. The level of confusion of each patient at that mealtime was determined from the ward nurse responsible for their care and recorded as follows: 0 = none, 1 = mild, 2 = moderate, 3 = severe, 9 = unknown. Ward staffing levels were recorded. On dietary study days ward-level balance data were also collected: all food delivered to the wards at mealtimes was documented and uneaten food was pooled and frozen for analysis of its nitrogen and energy content.

#### 2. Clinical outcomes data collection

Patients were recruited in a prospective consecutive manner over the two years of the study for collection of clinical outcomes data, and written informed consent was obtained. If a patient was unable to give consent the next of kin were asked if routinely collected clinical data could be recorded to allow the inclusion of patients who lacked mental capacity in the study group.

##### Data collected on admission

Demographic information including diagnoses, co-morbidities, geriatric syndromes, medication and usual living arrangements were abstracted from the medical records, as well as routinely collected clinical data including nutritional status (weight, BMI and ‘MUST’ score), cognitive function (Abbreviated Mental Test Score (AMTS) [28] and standard blood tests (full blood count, renal, liver and thyroid function tests, blood glucose, C reactive protein).

Body composition was assessed using anthropometry (mid upper arm circumference and triceps skinfold thickness of right arm) [29] and multi-frequency bioelectrical impedance (Impedimed; Queensland Australia). Maximal voluntary grip strength was measured 3 times in each hand using a Jamar dynamometer (Lafayette Instrument Company, USA) and a standard protocol [30].

Physical function was assessed using the Barthel Score [31]; a number of patients also had their mobility assessed using a small activity monitor (ActivPAL™) attached to their leg [32]. Cognitive function was further assessed using the Mini Mental State Examination (MMSE) [33]. Folic acid, vitamin B12 and Vitamin D levels were measured at entry to the study and at hospital discharge where these time points were at least 2 weeks apart.

##### Data collected on discharge

At discharge the type of domicile and care provided was recorded, along with changes in medication and any hospital acquired infections or complications. Anthropometry, bioimpedance, grip strength, Barthel score and ‘MUST’ score were repeated. Patient and/or carer satisfaction with hospital stay and specifically meals and mealtimes were assessed for all patients prior to discharge using the NHS catering satisfaction survey published by the Better Hospital Food Panel. The Warwick-Edinburgh Mental Well-being Scale was also administered at this point [34]. Staffing number, seniority and multi-disciplinary range was recorded weekly.

##### Data collected six and twelve months after discharge

Participants’ General Practitioners gave permission for a research nurse to contact the patient prior to either a home visit or a telephone call. Any change in the type of domicile, care provided or medication was recorded, as well as the number of readmissions and further contacts with healthcare professionals, or any deaths. Anthropometry, grip strength, Barthel Score and MMSE were repeated on the home visits.

#### 3. Qualitative data collection

A qualitative researcher conducted semi-structured interviews in both years with a purposive sample of 15–20 patients, or relatives/carers in cases where the patient lacked the capacity to participate, drawn from the intervention and control ward and selected to provide a breadth of views and experience.

Small group and individual semi-structured interviews were held with a range of senior and junior staff drawn from each ward in both years to elicit their views and experience of providing nutritional care for older people in hospital. A series of focus groups were held with volunteers in the second year to explore their experience of providing mealtime assistance on the wards. Both the semi–structured interview schedule and the focus group topic guide were developed with the hospital catering user group, which included service users and staff. They were refined to allow themes emerging from early interviews to be explored in subsequent interviews.

##### Sample size

Sample size calculations were based on a change in energy intake resulting from the intervention. A sample size of 100 subjects per ward per year is estimated to be sufficient to detect a difference in intake of 218 kcal/day with 80% power and p = 0.05, based on a mean (SD) intake of 1300 (550) kcal/day.

##### Statistical analysis

Descriptive statistics will be used to describe the participants during both years of the study, in conjunction with statistical tests for differences in characteristics between participants in the two wards in the baseline year. Descriptive statistics will also be used to describe the characteristics and mealtime duties of the volunteer mealtime assistants.

Differences in food and nutrient intake will be evaluated between patients on the intervention and control wards, and before and after the mealtime assistance on the intervention ward. Analyses, using Stata release 11, will consider differences between wards and year one and two, including planned subgroup analysis of dietary intake examining patients who required assistance, those who had soft diets, and those who were malnourished. In addition to standard descriptive statistics, a General Linear Model will be used to compare wards and time periods, and the analysis will include analysis of covariance.

The patient/carer and staff interviews and the volunteer focus groups were audio recorded with express consent from the participants and the audiofiles were transcribed verbatim. The data will be managed using a systematic framework approach facilitated by the software package NVivo 9 and analysed thematically. Two researchers will work separately and together to agree on key issues for the analytic framework [35], based on close reading of the transcripts, and will work through the data to draw out the full range of experiences and views, looking for commonality and differences within and between the participants.

##### Progress to date

Trained volunteer mealtime assistants have been working on the intervention ward since February 2011. They have been well received by patients and staff, and are enjoying their role. Dietary data collection has been completed for both years, totalling 222 patients’ food diaries in year one and 185 food diaries in year two. Clinical outcomes data have been collected during the admissions of 205 patients in year one and 137 patients in year two, and the follow-up visits are on-going. The interviews and focus groups have taken place in both year one and in year two, and analysis of this data is underway.

## Discussion

Poor nutrition of older people in hospital is an issue of concern in the UK and many other countries and is associated with major adverse clinical outcomes. The use of volunteer mealtime assistants has been advocated as a means of improving the nutrition of older in-patients but there is currently little evidence that they improve dietary intake or clinical outcomes. This study will measure dietary intake in older patients before and after the introduction of trained volunteer mealtime assistants onto an acute medicine for older persons ward and evaluate their effectiveness at increasing intake among patients on the intervention ward by comparison with contemporaneous data collection on patients in a control ward. The association of dietary intake and clinical outcomes including malnutrition risk, body composition, grip strength, length of stay and mortality will be assessed. Qualitative data will provide valuable data on the views, experience and satisfaction of patients, carers, staff and volunteers with nutrition in hospital and the impact of mealtime assistance.

## Abbreviations

MRC: Medical Research Council; NIHR: National Institute for Health Research; NHS: National Health Service; UK: United Kingdom; ‘MUST': ‘Malnutrition Universal Screening Tool’; BMI: Body Mass Index; AMTS: Abbreviated Mental Test Score; MMSE: Mini Mental State Examination; SD: Standard Deviation.

## Competing interests

The authors declare that they have no competing interests.

## Authors’ contributions

HR contributed to study design, co-ordinated and supervised data collection, analysed qualitative data and drafted the manuscript. AP participated in dietary data collection and analysis and assisted with drafting the manuscript. ME, AJ and CC contributed to study design and coordination and helped draft the manuscript. AAS conceived of the study, contributed to study coordination and helped draft the manuscript. SMR conceived of the study, collected and analysed dietary data and helped draft the manuscript. All authors read and approved the final manuscript.

## Pre-publication history

The pre-publication history for this paper can be accessed here:

http://www.biomedcentral.com/1471-2318/13/5/prepub
